# Neurogenic pulmonary edema secondary to epileptic seizure

**DOI:** 10.1002/ccr3.3196

**Published:** 2020-08-04

**Authors:** Jun Yonekawa, Shinichi Miyazaki, Toshiaki Ieda, Takuya Ikeda

**Affiliations:** ^1^ Department of General Internal Medicine Yokkaichi Municipal Hospital Yokkaichi‐shi Japan; ^2^ Department of Respiratory Medicine Yokkaichi Municipal Hospital Yokkaichi‐shi Japan; ^3^ Department of Neurology Yokkaichi Municipal Hospital Yokkaichi‐shi Japan

**Keywords:** epileptic seizure, neurogenic pulmonary edema

## Abstract

Neurogenic pulmonary edema (NPE) is a non‐cardiogenic pulmonary edema that is caused by an acute central nervous system injury and usually develops rapidly after an injury. Although several episodes of NPE resolve spontaneously, the condition may cause unexpected death among patients with epilepsy.

## Graphical abstract

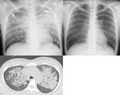

## 1 CASE DESCRIPTION

**FIGURE 1:**
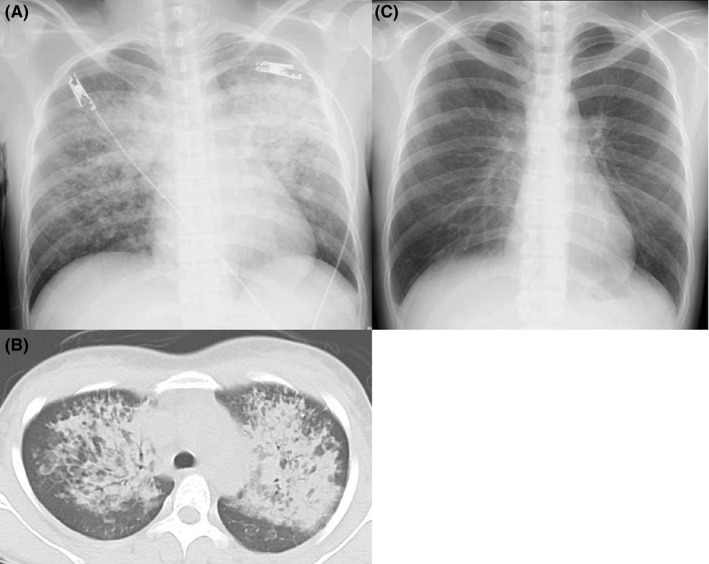
A, Chest radiography image upon admission, (B) computed tomography of the chest upon admission, (C) and chest radiography 4 d after admission

A 29‐year‐old woman presented to the emergency department 30 minutes after experiencing two episodes of tonic‐clonic seizures, each lasting < 5 minutes, and she had full recovery of consciousness with no respiratory symptoms. The patient had her first episode of seizure 6 years before presentation. However, anti‐seizure drug therapy was not started because of low risk of recurrence. Upon examination, the patient was afebrile. Her respiratory rate was 33 breaths per minute and oxygen saturation was 74% on room air, which was recovered to 92% after administering 10 L/min of oxygen via mask with a reservoir bag. Other physical examinations were normal. Chest radiography (Figure 1A) and computed tomography (CT) (Figure 1B) revealed bilateral consolidation with a central distribution and sparing of the lung cortex. Electrocardiogram and laboratory examination results were normal, and brain CT revealed no intracranial lesions.

Furthermore, without additional treatment, the patient's clinical condition rapidly normalized over the next few hours, and her oxygen saturation was 97% on oxygen supplementation at 3 L/min via nasal cannula. She was off oxygen therapy within 24 hours, and repeat chest radiograph conducted after 4 days was normal (Figure 1C). She was then discharged after the initiation of antiepileptic medication.

Neurogenic pulmonary edema (NPE) is a non‐cardiogenic pulmonary edema that occurs after a significant central nervous system insult. Although several episodes of NPE are well tolerated, the condition may cause unexpected death among patients with epilepsy.^1^

## CONFLICT OF INTEREST

The authors have no conflicts of interest.

## AUTHOR CONTRIBUTIONS

JY: involved in patient management and wrote the manuscript. SM and TI: helped in the preparation of the manuscript. TI: contributed to the critical review of the manuscript.

## INFORMED CONSENT

Informed consent was obtained from the patients prior to conducting this study.
